# Changes in mode of travel to work: a natural experimental study of new transport infrastructure

**DOI:** 10.1186/s12966-015-0239-8

**Published:** 2015-06-20

**Authors:** Eva Heinen, Jenna Panter, Roger Mackett, David Ogilvie

**Affiliations:** MRC Epidemiology Unit and UKCRC Centre for Diet and Activity Research (CEDAR), School of Clinical Medicine, University of Cambridge, Cambridge Biomedical Campus, Box 285, Cambridge, CB2 0QQ UK; Department of Civil, Environmental and Geomatic Engineering, University College London, Gower Street, London, WC1E 6BT UK

**Keywords:** Adults, Active travel, Bus, Commuting, Evaluation, Intervention, Natural experimental study, Transport infrastructure, Travel behaviour, Modal shift

## Abstract

**Background:**

New transport infrastructure may promote a shift towards active travel, thereby improving population health. The purpose of this study was to determine the effect of a major transport infrastructure project on commuters’ mode of travel, trip frequency and distance travelled to work.

**Methods:**

Quasi-experimental analysis nested within a cohort study of 470 adults working in Cambridge, UK. The intervention consisted of the opening of a guided busway with a path for walking and cycling in 2011. Exposure to the intervention was defined as the negative of the square root of the shortest distance from home to busway. The outcome measures were changes in commute mode share and number of commute trips — both based on a seven-day travel-to-work record collected before (2009) and after (2012) the intervention — and change in objective commute distance. The mode share outcomes were changes in the proportions of trips (i) involving any active travel, (ii) involving any public transport, and (iii) made entirely by car. Separate multinomial regression models were estimated adjusting for commute and sociodemographic characteristics, residential settlement size and life events.

**Results:**

Proximity to the busway predicted an increased likelihood of a large (>30 %) increase in the share of commute trips involving any active travel (relative risk ratio [RRR] 1.80, 95 % CI 1.27, 2.55) and a large decrease in the share of trips made entirely by car (RRR 2.09, 95 % CI 1.35, 3.21), as well as a lower likelihood of a small (<30 %) reduction in the share of trips involving any active travel (RRR 0.47, 95 % CI 0.28, 0.81). It was not associated with changes in the share of commute trips involving any public transport, the number of commute trips, or commute distance.

**Conclusions:**

The new infrastructure promoted an increase in the share of commuting trips involving active travel and a decrease in the share made entirely by car. Further analysis will show the extent to which the changes in commute mode share were translated into an increase in time spent in active commuting and consequent health gain.

**Electronic supplementary material:**

The online version of this article (doi:10.1186/s12966-015-0239-8) contains supplementary material, which is available to authorized users.

## Introduction

Active travel can provide a sufficient level of physical activity to improve health and well-being [1]. This activity may be particularly beneficial in the form of active commuting, which offers the potential to easily incorporate regular walking and cycling into daily life [2]. However, not all environments are equally supportive for walking and cycling. Although cross-sectional studies show that characteristics of the built environment are associated with differences in travel behaviour, including walking and cycling [3–7], few intervention studies exist in this area [8–10] and this limits the possibility of determining the causal effects of environmental changes on travel behaviour [11–13].

The primary outcome of most interest to health researchers studying interventions in this area is often a change in time spent in active travel [14, 15]. However, understanding the population health impacts of infrastructural interventions may require an approach that considers a wider range of outcomes [16]. Change in time spent in active travel may be a result of changes in the distance, speed, and number of trips and in the proportions of these made by each mode of transport (mode share). The patterns and determinants of changes in these characteristics may differ, and they may also have different long-term health consequences. For example, a reduction in car travel could confer population health benefits including reduced exposure to air pollution and injuries, while an increase in walking or cycling could confer individual health benefits resulting from increased physical activity [17].

The natural experimental study [18] ‘Commuting and Health in Cambridge’ offers an opportunity to study the effect of new transport infrastructure on travel behaviour [19]. It investigates the introduction of a guided busway, which is the longest of its kind in the world and is unique in providing high-quality facilities for three modes of transport: bus, cycling and walking. Building on existing intervention studies, this study explores changes in travel behaviour in more depth by examining multiple outcomes. Specifically, this paper aims to determine the effect of the intervention on changes in mode share, number of trips and distance travelled on the journey from home to work.

## Methods

### Intervention: the Cambridgeshire Guided Busway

The busway is situated in Cambridgeshire, UK. The city of Cambridge has a comparatively affluent and well-educated population. 45 % of its commuting population travel to work by car or taxi, 28 % by bicycle, 15 % on foot and 9 % by public transport [20].

**Fig. 1:**
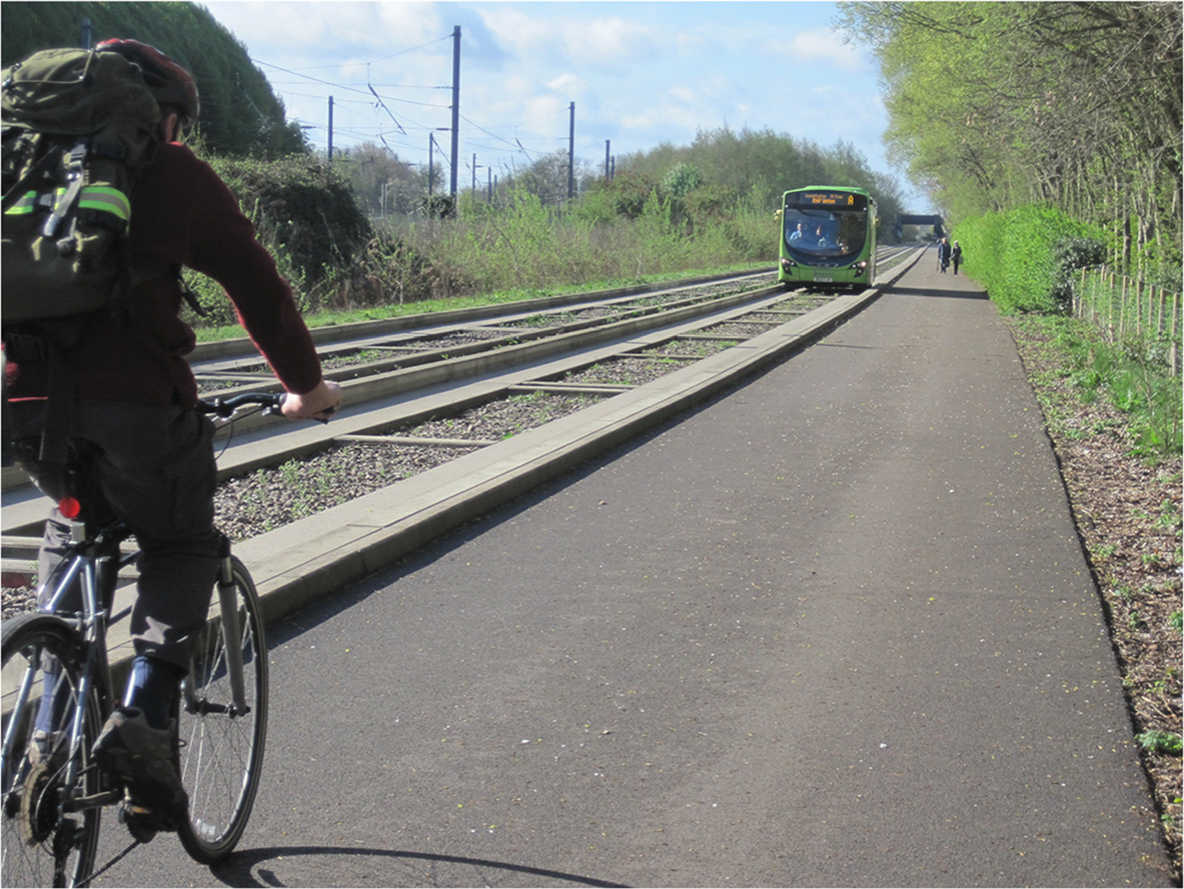
The Cambridgeshire Guided Busway

The busway comprises a 25 km off-road guideway for buses, with a parallel path that can be used for walking and cycling (Fig. 1), in two sections: one between the market town of St Ives and the northern edge of Cambridge, and the other between Cambridge railway station and the southern fringe at Trumpington. The busway links surrounding villages, the urban fringe, the city centre and several major employment sites [21]. Opened in 2011, it offers better than average facilities for bus use, cycling and walking and was intended to change travel behaviour in order to reduce traffic congestion [22, 23].

### Study sample

Questionnaire data were collected by post annually between 2009 and 2012 as part of a natural experimental cohort study. Participants, who were recruited mainly through workplaces, were aged 16 or over, working in areas of Cambridge to be served by the busway and living within approximately 30 km of the city centre [19]. To avoid biasing recruitment and responses, the study was presented to participants as a study of ‘commuting and health’ and the aim of evaluating the busway was not made explicit.

In this paper we analyse the first (pre-intervention) and fourth (final, post- intervention) survey waves. 1164 participants took part in the first wave, of whom 500 (43 %) also took part in the fourth wave.

The Hertfordshire Research Ethics Committee approved the study and the baseline data collection (reference number: 08/H0311/208) and the Cambridge Psychology Research Ethics Committee granted approval for the follow-up data collection used in this analysis (reference number: 2014.14). All participants provided written informed consent.

### Outcomes

Three outcomes were modelled: change in commute mode share, change in number of commute trips and change in commute distance.

The first two outcomes were derived from a seven-day commute travel diary [24]. For each day, respondents were asked to report the day of the week, their working hours and their mode(s) of travel to and from work, or to positively indicate that they had not travelled to work that day. We excluded respondents who had returned a blank travel diary in either wave (*n* = 28) or had accounted for fewer than three days of the week (*n* = 2), leaving 470 respondents included in analysis. We truncated the travel diary to the first seven reported consecutive days if more days were reported. Individuals reporting fewer or more than seven consecutive days, failing to report an apparently missing trip to or from work (unless at the beginning or end of the reporting period), or failing to report the day of the week were included in the main analysis but excluded from a subsequent sensitivity analysis (S4, see below). No imputations were made if travel data appeared incomplete. We derived three specific mode share outcomes. These were changes in the share of trips (i) involving any active travel, (ii) involving any public transport, and (iii) made entirely by car, reflecting the aims and nature of the intervention.

Change in objective commute distance was determined using self-reported home and workplace postcodes and calculated using a geographic information system (GIS) (Additional file 1).

### Exposure

We derived an objective, ego-centred [25] measure of exposure to the intervention for each individual, based on the proximity of their baseline home postcode to the nearest busway stop or path access point. We recognised that use of the busway was unlikely to be limited to a specific distance buffer, which meant that assigning individuals to simple ‘intervention’ and ‘control’ groups based on area of residence would not be meaningful [18]. Preliminary descriptive analyses also showed statistically significant differences in some covariates between different parts of the study area, which reinforced the decision to treat exposure to the intervention as a continuous variable. We expected a given increment in distance to have a smaller effect on travel behaviour as distance increased, and therefore defined exposure using a square root transformation of the negative of the distance (hence shorter distance = greater proximity = greater exposure). This produced comparable, but slightly more conservative and more easily interpretable, model outputs to those produced by a log transformation, the most obvious alternative.

### Covariates

We controlled for alternative explanations such as life events which have been shown to be associated with travel behaviour change. Changes in home or work location could affect travel behaviour [26–28] and were operationalized for analysis as change in objective commute distance,^1^ change in estimated travel time to work by public transport, and change in distance from home to the busway. Further details are provided in Additional file 1. We also controlled for two additional changes: a change in workplace parking provision [29] and a change in the number of children in the household [26].

The following additional covariates were included: gender, age, commute distance, the availability of (free) parking at work, education level, car ownership, access to a bicycle, presence of children in the household, presence of a limiting long-term health condition, difficulty walking, the mental (MCS-8) and physical (PCS-8) summary scores of the SF-8 [30] and residential settlement size (the Urban Rural Classification of the Census Output Area) [31], all assessed at baseline (Table 1). We tested for interaction effects between exposure to the intervention and commute distance, gender, change in estimated travel time to work by public transport, and change in commute distance. We calculated the interaction terms by multiplying the mean-adjusted values of two continuous variables with each other, or in case of a nominal or ordinal variable, the mean-adjusted value if a condition was met (i.e., male =0; female = value).

**Table 1:** Summary of participant characteristics, baseline travel behaviour and exposure to intervention

		All participants at wave 1				Analysis sample (valid wave 1 and wave 4)				Participants in wave 1, but not wave 4 (drop-out)^a^			
		n	%	mean	st.d.	n	%	mean	st.d.	n	%	mean	st.d.
Distance from home to busway (km)		1155		6.6	7.8	466		6.5	7.9	659		6.7	7.7
Proximity to busway (-√km)		1155		−2.2	1.4	466		−2.1	1.4	659		−2.2	1.4
Commute distance (km)		1158		11.1	9.4	469		10.9	9.4	659		11.4	9.5
Change in commute distance (km)						450		0.2	4.9				
Change in travel time to work by public transport (min)						443		−0.1	11.1				
Change in proximity to busway (km)						450		0.2	4.9				
Moved home	No					359	76.4						
	Yes					111	23.6						
Moved workplace	No					357	76						
	Yes					113	24						
Gender	Male	367	31.5			157	33.4			205	30.9		
	Female	797	68.5			313	66.6			459	69.1		
Age	≤30	197	17			58	12.4			135	20.4		
	31–40	327	28.2			111	23.7			207	31.3		
	41–50	305	26.3			139	29.6			160	24.2		
	51–60	246	21.2			122	26			114	17.2		
	61+	85	7.3			39	8.3			46	6.95		
Education level	Degree	837	72.3			350	74.6			471	71.4		
	Less than degree	321	27.7			119	25.4			189	28.6		
Housing tenure	Not owner	319	27.5			103	22			204	30.9		
	Owner	840	72.5			365	78			457	69.1		
Driving licence	No	113	9.7			37	7.9			72	10.9		
	Yes	1049	90.3			432	92.1			591	89.1		
Access to a bicycle	No	182	15.7			63	13.5			191	28.8		
	Yes	974	84.3			404	86.51			473	71.2		
Children in household	No	820	70.5			324	68.9			473	71.2		
	Yes	344	29.6			146	31.1			191	28.8		
New child in household	No					444	94.5						
	Yes					26	5.5						
Physical health (PCS-8)		1156		53.7	6.3	468		53.9	6.3	658		53.6	6.4
Mental health (MCS-8)		1156		50.6	8.1	468		51.7	7.1	658		49.9	8.6
Limiting health condition	No	1040	89.7			429	91.7			582	88.1		
	Yes	119	10.3			39	8.3			79	12		
Difficulty walking	No	1143	98.5			464	98.9			651	98.3		
	Yes	18	1.6			5	1.1			11	1.7		
Type of settlement	Urban (>10,000)	767	66			316	67.4			427	64.3		
	Town & Fringe	226	19.4			80	17.1			144	21.7		
	Village, Hamlet & Isolated Dwellings	170	14.6			73	15.6			93	14.0		
Car parking at work	No	371	32.3			151	32.4			208	31.8		
	Yes, paid	351	30.6			143	30.7			201	30.7		
	Yes, free	427	37.2			172	36.9			245	37.5		
Change in car parking at work from parking to no parking	No					405	86.2						
	Yes					65	13.8						
Car ownership	No car	175	15.0			57	12.1			112	16.9		
	One car	525	45.1			225	47.9			287	43.2		
	Two or more cars	464	39.9			188	40.0			265	39.9		
Baseline proportion of trips involving any active travel^b^		1141		0.67	0.43	467		0.69	0.42	648		0.65	0.44
Baseline proportion of trips involving any public transport^b^		1141		0.15	0.32	467		0.15	0.32	648		0.14	0.32
Baseline proportion of trips made entirely by car^b^		1141		0.27	0.4	467		0.24	0.38	648		0.29	0.42
Baseline number of commute trips^c^		1164		9.2	2.8	470		9.3	2.6	664		9.2	2.8

^a^Significant differences were found between the analysis sample and those excluded owing to dropout or other exclusion criteria in age, housing tenure, MCS-8, presence of a limiting health condition, and baseline car commute share

^b^If no trips had been made, the mode share was coded as missing

^c^Including reports of zero trips where these were deemed to be true zeroes rather than missing values

### Analyses

Changes in mode share were grouped into five categories corresponding to a large (30 % to 100 %) decrease, a small (<30 %) decrease, no change, a small (<30 %) increase and a large (30 % to 100 %) increase, except for the change in public transport mode share which was grouped into three categories (decrease, no change and increase) due to its distribution. A change of 30 % (i.e., three trips out of ten) or more was considered a substantial change as it corresponds in most cases to a change affecting more than one day per week. Change in the weekly number of trips was similarly grouped into five categories: large decrease (3 trips or more), small (<3 trips) decrease, no change, small (<3 trips) increase and large increase (3 trips or more) (Table 2).

**Table 2:** Distributions of main outcome variables

		Number	Percent	Mean	st.d.
Change in proportion of trips involving any active travel	Decrease of 30 % or more	58	12.9		
	Decrease of <30 %	36	8.0		
	No change	276	61.2		
	Increase of <30 %	34	7.5		
	Increase of 30 % or more	47	10.4		
Change in proportion of trips involving any public transport	Decrease	55	12.2		
	No change	341	75.6		
	Increase	55	12.2		
Change in proportion of trips made entirely by car	Decrease of 30 % or more	31	6.9		
	Decrease of <30 %	39	8.7		
	No change	286	63.4		
	Increase of <30 %	42	9.3		
	Increase of 30 % or more	53	11.8		
Change in number of trips	Decrease of 3 trips or more	87	18.6		
	Decrease of <3 trips	77	16.4		
	No change	194	41.4		
	Increase of <3 trips	62	13.2		
	Increase of 3 trips or more	49	10.5		
Change in commute distance (km)		450		0.20	4.9

Effects on mode share and number of trips were tested with multivariable multinomial logistic regression models, progressively adjusted as follows: (1) unadjusted, (2) adjusted for commute characteristics, (3) adjusted for commute and sociodemographic characteristics, and (4) maximally adjusted for commute, sociodemographic and spatial characteristics. Age and gender were always included, and other explanatory variables associated with the outcome at *p* < 0.25 in unadjusted models were included in the adjusted models [32]. Interaction effects were included only if significant at *p* < 0.05. None of the interaction effects met that condition.

Four sensitivity tests were conducted on each maximally adjusted model: (S1) including the baseline value of the outcome (mode share or number of trips as appropriate) as a continuous independent variable; (S2) including workplace parking and car ownership; (S3) restricted to individuals who did not move home or workplace between baseline and follow-up; and (S4) restricted to individuals who had ‘perfectly’ completed travel diaries at both time points (see above). Additional sensitivity tests restricting the analyses to individuals who reported more than four or more than six trips showed no impact on the effect estimates (data not shown).

## Results

Of the 470 participants included in analysis, 175 reported a change in their active travel mode share for commuting (average change of −1.3 %), 110 a change in their public transport mode share (average change −1.0 %) and 165 a change in their car mode share (average change +3.4 %).

### Changes in commute mode share

#### Trips involving any active travel

Proximity to the busway was significantly associated with a small decrease in active travel mode share in unadjusted analyses, but in the maximally adjusted models proximity predicted a large increase in active travel mode share (relative risk ratio [RRR] 1.80, 95 % confidence interval [95 % CI] 1.27 to 2.55) and reduced the likelihood of a small decrease in active travel mode share (RRR 0.47, 95 % CI 0.28 to 0.81) (Table 3 and Additional file 2). In other words, commuters living 4 km from the busway were almost twice as likely to report a substantial increase in their active travel mode share, and half as likely to report a small decrease, than those living 9 km away. Both associations were strengthened in analyses restricted to participants who did not move home or workplace.

**Table 3:** Associations between exposure to busway and changes in active travel mode share

Outcome	Change in active travel mode share			
	Large decrease	Small decrease	Small increase	Large increase
Unadjusted	0.94 (0.77, 1.14)	0.77 (0.62, 0.97)*	0.99 (0.77, 1.27)	1.15 (0.90, 1.46)
Adjusted for commute characteristics	1.09 (0.80, 1.47)	0.57 (0.35, 0.92)*	0.63 (0.37, 1.06)	1.53 (1.14, 2.06)**
Adjusted for commute and sociodemographic characteristics	1.04 (0.75, 1.44)	0.47 (0.28, 0.81)**	0.64 (0.35, 1.15)	1.57 (1.14, 2.15)**
Maximally adjusted model	1.08 (0.77, 1.50)	0.47 (0.28, 0.81)**	0.69 (0.38, 1.26)	1.80 (1.27, 2.55)**
Sensitivity tests:				
S1. Including baseline	1.07 (0.74, 1.54)	0.48 (0.28, 0.81)**	0.70 (0.38, 1.27)	1.71 (1.15, 2.55)**
S2. Including parking	1.19 (0.85, 1.68)	0.47 (0.27, 0.81)**	0.68 (0.36, 1.28)	1.94 (1.34, 2.80)**
S3. Non-movers only	1.06 (0.68, 1.65)	0.31 (0.10, 0.94)*	0.79 (0.36, 1.70)	1.84 (1.16, 2.90)**
S4. Perfect diaries only	1.26 (0.85, 1.88)	0.35 (0.17, 0.73)*	0.58 (0.27, 1.25)	1.80 (1.20, 2.70)**

Multinomial logistic regression with ‘no change’ as the reference outcome category

Exposure to the busway was defined as the negative square root of the distance from home to busway

Values tabulated are relative risk ratios (95 % confidence intervals)

*p < 0.05; **p < 0.01

In addition, home ownership reduced the likelihood of a small decrease in active travel mode share, whereas those aged 51–60 were more likely to report a large decrease than those aged 31–40. Commuters with a child in their household were more likely to report a small increase in active travel mode share, whereas a large increase was more likely among those whose commute distance had increased or who lived in very small settlements and less likely among those with degree-level education.

#### Trips involving any public transport

As expected, in the unadjusted model commuters living closer to the busway were less likely to reduce their public transport mode share, but this effect became insignificant after adjustment for commute characteristics and remained insignificant after further adjustment (Table 4 and Additional file 3).

**Table 4:** Associations between exposure to busway and changes in public transport mode share

Outcome	Change in public transport mode share	
	Decrease	Increase
Unadjusted	0.82 (0.68, 0.99)*	0.94 (0.77, 1.14)
Adjusted for commute characteristics	0.90 (0.67, 1.20)	1.07 (0.82, 1.41)
Adjusted for commute and sociodemographic characteristics	0.92 (0.68, 1.25)	1.16 (0.86, 1.55)
Maximally adjusted model	0.91 (0.66, 1.24)	1.26 (0.92, 1.72)
Sensitivity tests:		
S1. Including baseline	0.93 (0.63, 1.37)	
S2. Including parking	0.92 (0.66, 1.27)	1.28 (0.93, 1.76)
S3. Non-movers only	0.99 (0.66, 1.49)	1.30 (0.91, 1.86)
S4. Perfect diaries only	1.03 (0.71, 1.48)	1.43 (1.00, 2.05)

Multinomial logistic regression with ‘no change’ as the reference outcome category

Exposure to the busway was defined as the negative square root of the distance from home to busway

Values tabulated are relative risk ratios (95 % confidence intervals)

*p < 0.05; **p < 0.01

Several covariates were significantly associated with a change in public transport mode share. Having a bicycle or higher self-rated physical health (PCS-8) reduced the likelihood of a decrease in public transport mode share, whereas living in villages or smaller settlements rather than urban areas predicted an increase in public transport mode share.

#### Trips made entirely by car

Proximity to the busway did not predict changes in car mode share in unadjusted analyses, but after adjustment for commute characteristics and all further adjustments it was associated with a large decrease in car mode share (RRR 2.09, 95 % CI 1.35 to 3.21) (Table 5 and Additional file 4). This effect was robust to the sensitivity analyses, and corresponds to a doubling of the likelihood of a large decrease in car use between those living 9 km from the busway and those living 4 km away.

**Table 5:** Associations between exposure to busway and changes in car mode share

Outcome	Change in car mode share			
	Large decrease	Small decrease	Small increase	Large increase
Unadjusted	1.16 (0.86, 1.57)	0.85 (0.68, 1.07)	0.82 (0.66, 1.01)	0.86 (0.70, 1.05)
Adjusted for commute characteristics	1.71 (1.20, 2.44)**	0.60 (0.37, 0.97)*	0.70 (0.45, 1.07)	1.13 (0.85, 1.51)
Adjusted for commute and sociodemographic characteristics	1.71 (1.17, 2.49)**	0.59 (0.36, 0.99)*	0.65 (0.41, 1.03)	1.08 (0.79, 1.48)
Maximally adjusted model	2.09 (1.35, 3.21)**	0.64 (0.38, 1.08)	0.71 (0.44, 1.14)	1.10 (0.80, 1.52)
Sensitivity tests:				
S1. Including baseline	2.57 (1.47, 4.49)**	0.65 (0.38, 1.11)	0.71 (0.44, 1.13)	1.03 (0.73, 1.44)
S2. Including parking	2.39 (1.47, 3.91)**	0.61 (0.33, 1.14)	0.72 (0.45, 1.15)	1.17 (0.84, 1.64)
S3. Non-movers only	1.84 (1.06, 3.21)*	0.71 (0.37, 1.38)	0.59 (0.29, 1.20)	1.10 (0.72, 1.68)
S4. Perfect diaries only	2.17 (1.30, 3.62)**	0.52 (0.26, 1.03)	0.75 (0.40, 1.40)	1.13 (0.76, 1.68)

Multinomial logistic regression with ‘no change’ as the reference outcome category

Exposure to the busway was defined as the negative square root of the distance from home to busway

Values tabulated are relative risk ratios (95 % confidence intervals)

*p < 0.05; **p < 0.01

Furthermore, an increase in commute distance was associated with a small decrease in car mode share, while those with children or living in less urban settlements were more likely to report a large decrease in car mode share. Commuters aged 51–60 were more likely to report a large increase in car mode share, whereas those with higher self-rated mental health (MCS-8) were less likely to do so.

### Change in number of commute trips

Proximity to the intervention did not predict a change in number of trips (Additional file 5). Women were more than twice as likely as men to report a large decrease in trips, those aged 41–50 were less likely than those aged 31–40 to report a small increase, and those with a degree were more likely to report a large increase.

### Change in commute distance

While the intervention reduced the distance from home to work on foot for 55 individuals (mean change -42 m, s.d. 173 m), the majority of individuals experiencing a change in distance had moved home or workplace. Proximity to the busway did not predict a reduction in commute distance that was not due to moving (p > 0.25, data not shown).

## Discussion

### Principal findings

Our findings provide new evidence linking new transport infrastructure with travel behaviour change. After adjusting for multiple potential confounders, exposure to the intervention (proximity to the busway) was significantly associated with the likelihood of making large changes in commute mode choice. Individuals who lived closer to the busway were more likely to report a large increase in the proportion of their commute trips that involved active travel than those who lived further away. Exposure to the intervention also predicted a large decrease in the share of commute trips made entirely by car and ‘prevented’ against a small decrease in the share of commute trips involving active travel.

### Interpretation

The busway was intended to increase public transport use, mainly by attracting car users [33]. We found no significant effect on overall public transport mode share in adjusted models, although the differences observed were in the expected direction. This may reflect the relatively low proportion of public transport trips in our sample at baseline, and the fact that shifts from conventional bus to guided bus services would not have been captured by this measure. The high local prevalence of cycling may explain why this particular intervention showed a clearer effect on active travel in our sample, and the shift in commute mode share from the car towards active travel may also have contributed to a desirable reduction in traffic congestion. Furthermore, since the intervention was not associated with changes in commute distance or trip frequency, it is plausible that this modal shift may be translated into an increase in time spent in active travel and consequent health benefits.

For certain outcomes the intervention effect became significant only after adjustment for covariates, which indicates that the effect was suppressed by the commute and socioeconomic characteristics of the participants. This probably reflects the fact that exposure to the intervention was not evenly distributed in relation to those characteristics, and the statistical relationship between exposure and behaviour change was therefore masked by the correlation between exposure and the covariates.

Travel behaviour change was more strongly associated with the intervention than with moving or becoming a parent. Controlling for other changes unrelated to the intervention eliminated several plausible alternative explanations for the changes in travel behaviour. Moreover, the sensitivity analyses restricted to participants who had not moved or experienced a change in workplace parking provision confirmed or even strengthened the associations observed in the main analyses, indicating that behaviour change can occur independently of major life events.

For some covariates, the associations differed from those typically observed in cross-sectional studies. For example we found that an increase in commute distance corresponded with a small decrease in car commuting, while longer commute distances are more often associated with a higher likelihood of car commuting. One explanation for this is that individuals making greater use of a given mode of transport at baseline have a higher chance of reducing its use over time than those who never or rarely used it, and more generally it is important to note that correlates of change in behaviour over time are likely to differ from correlates of behaviour at a single time point.

### Strengths and limitations

The key strengths of this study include the use of an individually computed measure of intervention exposure, multiple measures of behaviour change outcomes and a large number of covariates to eliminate alternative causal explanations. Nevertheless, the study has a number of limitations. The first is that it was not possible to compare outcomes in intervention and control groups that differed only in their level of exposure to the intervention. However, natural experimental studies such as ours are often the only feasible intervention study design in this field. A second limitation is that travel behaviour was self-reported, which could threaten the validity of the outcome measures by intentional or unintentional misreporting. A third is that we applied a measure of exposure based solely on participants’ home addresses. Considering the working location and commute route would capture exposure more fully [25], and more detailed measures of individual exposure to environmental change may strengthen the evidence of intervention effects [9, 34]. However, our final choice of exposure measure followed a consideration of several alternatives, and we expected comparatively little variation in exposure by workplace because all respondents worked at locations that were at least notionally accessible using the busway.

### Implications

The intervention had a substantial influence on the proportion of commute trips that involved at least some active travel. This has the potential to improve health, whether directly or via higher levels of overall physical activity. Further longitudinal analysis will be necessary to determine whether these changes in mode share have resulted in increased physical activity and overall health gain.

The study investigated the effect of new infrastructure on travel behaviour only one year after it became fully operational. Travel mode choice is known to be habitual and strongly predicted by previous behaviour, which suggests that people may require more time to change their behaviour [35]. The longer-term effects of the intervention may therefore be larger or smaller than those observed in this analysis [8].

While the scale of this particular intervention made it a good subject for public health evaluation, opportunities to implement exactly the same type of intervention on a similarly large scale may be limited, and the findings of our cohort study may not be directly generalizable to other study populations and settings. However, this is true of much natural experimental research in public health [18]. The principles of the intervention and study design could nevertheless be generalized, and similar studies in different settings are necessary to build a body of evidence that is not specific to a single context. Such studies could corroborate or disconfirm our findings, and will contribute to the development of more conclusive evidence of a causal relationship between changes in the built environment and change in population activity patterns and health.

## Conclusion

Our findings show that the introduction of new transport infrastructure was associated with travel behaviour change. Over time, commuters with a higher level of exposure to the guided busway were more likely to have increased the proportion of their commute trips involving active travel, and to have reduced the proportion made entirely by car, than those with a lower level of exposure. The intervention was not associated with a change in the number or distance of commute trips, which suggests that the modal shift in commuting patterns may be translated into an increase in time spent in physical activity on the journey to and from work. These findings add to a growing body of evidence to support the assumption that changing the built environment can bring about changes in travel behaviour and contribute to consequent population health gain.

## Additional files

Additional file 1:
**Calculation of intervention exposure and commute distance and bus travel time [** 36 **–** 41 **].**
Additional file 2:
**Predictors of change in active travel mode share.**
Additional file 3:
**Predictors of a change in public transport mode share.**
Additional file 4:
**Predictors of change in car mode share.**
Additional file 5:
**Predictors of change in number of commute trips.**

## Footnotes

1 Except for the analysis in which change in commute distance was the outcome.
