# Supplementary material for: Changes in mode of travel to work: a natural experimental study of new transport infrastructure
Source: Int J Behav Nutr Phys Act. 2015 Jun 20;12:81. doi: 10.1186/s12966-015-0239-8 (PMC4496849; doi:10.1186/s12966-015-0239-8)
Supplement: Additional file 1: — Calculation of intervention exposure and commute distance and bus travel time [ 36 – 41 ]. [file 12966_2015_239_MOESM1_ESM.doc]

Additional file 1: Calculation of intervention exposure and commute distance and bus travel time

The exposure measures were calculated using geographic information system (GIS) software ArcGIS 9.3 and three created route networks: a car network (1) and two pedestrian and cyclist networks, reflecting the routes available before (2) and after (3) the intervention. These networks were created using the following data sources:

1. Car network: the Ordnance Survey (OS) MasterMap® Integrated Transport Network (ITN) [35-36] road network dataset was used, minus the busway itself which is not available for use by private motor vehicles and was therefore removed manually
2. Pre-intervention pedestrian and cyclist network: motorways and the busway path were removed from the car network (1), and footpaths and cycle routes were added from local authority data on rights-of-way (public footpaths, bridleways and byways) [37], cycle route information from the UK charity Sustrans [38] and any additional paths from OpenStreetMap.com [39].
3. Post-intervention pedestrian and cyclist network: as the pre-intervention network (2) but with the addition of the busway path.

Home and workplace postcodes reported in the questionnaire were georeferenced to a point on the ground using the OS CodePoint® database [40]. Distances between participants’ home and work locations before and after the intervention were calculated using the Network Analyst Route tool in ArcGIS and the pre- and post-intervention route networks. Distances between home and the nearest busway stop and the nearest access point to the busway path were calculated using the Network Analyst Closest Facility tool in ArcGIS, using the post-intervention pedestrian and cyclist network. Commute times by bus before and after the intervention were computed using Accession [41], a software package which uses road networks, road speed data and public transport timetable information to calculate travel times. This was used to estimate the quickest potential journey to work on a Wednesday, with the journey commencing after 6am and the work destination to be reached before 10am – a four hour period chosen to represent the peak morning commute.

The exposure data were cleaned to remove changes of <5m and merged with the socioeconomic and spatial data from the questionnaire. This allowed us to describe the characteristics of the sample and the sociospatial distribution of the exposure measures, and to confirm the choice of primary and secondary exposure measures while blinded to their relationship with the outcome measures. The data were then fully merged for the main analyses.
